# Supplementary material for: NO2 Sensing Behavior of Compacted Chemically Treated Multi-Walled Carbon Nanotubes
Source: Micromachines (Basel). 2022 Sep 8;13(9):1495. doi: 10.3390/mi13091495 (PMC9503782; doi:10.3390/mi13091495)
Supplement: Supplementary file 1 [file micromachines-13-01495-s001.zip › micromachines-1873968-supplementary.pdf]

micromachines

MDPI

Article

NO_2 sensing behavior of compacted chemically treated multi-walled carbon nanotubes

Nikita I. Lapekin[1], Valeriy V. Golovakhin[1], Ekaterina Yu. Kim[1] and Alexander G. Bannov[1,*]

[1] Department of Chemistry and Chemical Engineering, Novosibirsk State Technical University, 630073 Novosibirsk, Russia

* Correspondence: bannov.alexander@gmail.com

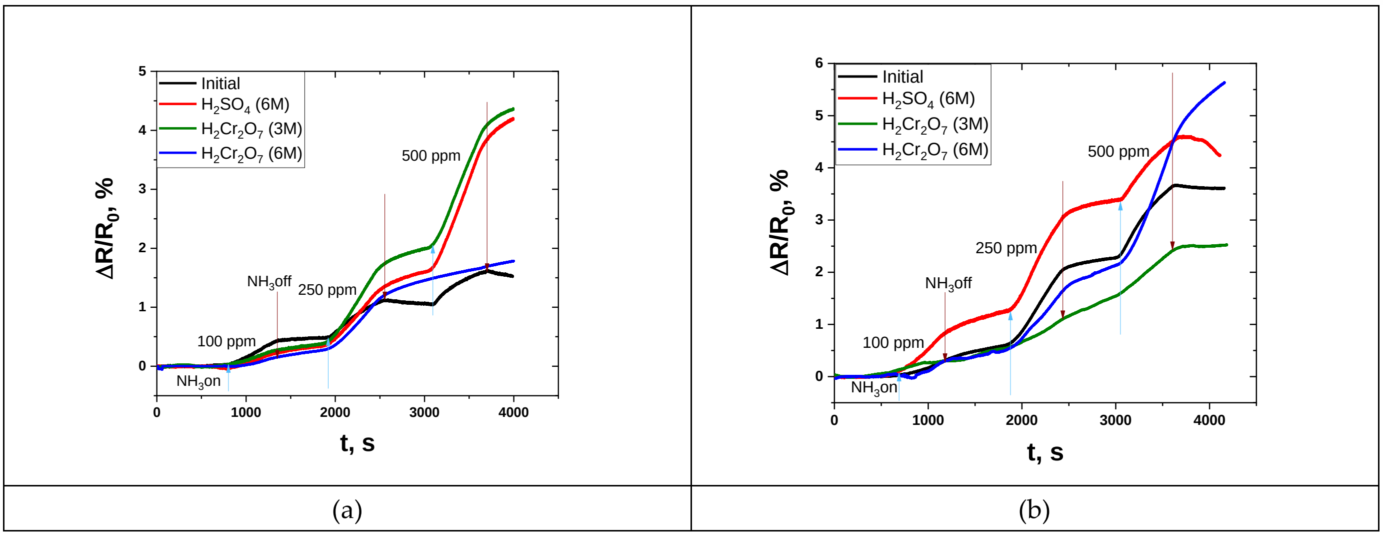

(a) (b)

Figure S1. Response of pure and treated MWNT-1020 (a) and MWNT-4060 (b) to 100-500 ppm NH_3 at room temperature (25±2°C)

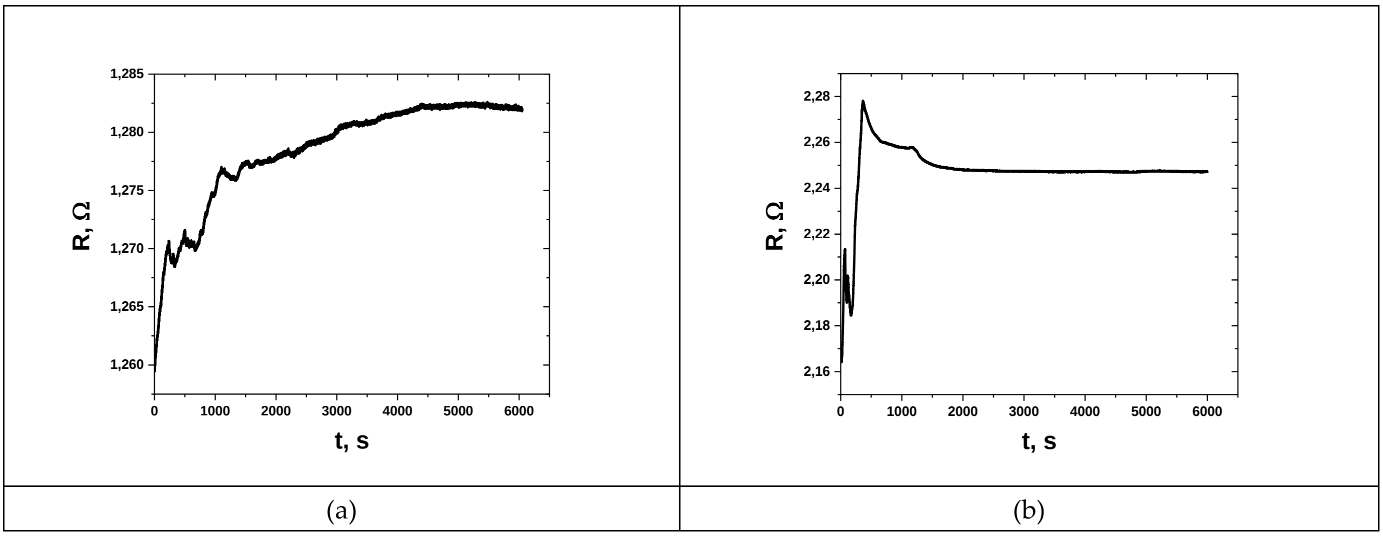

(a) (b)

Figure S2. Resistance of pure MWNT-1020 (a) and MWNT-4060 (b) to 100-500 ppm CH_4 at room temperature (25±2°C)
